# Associations between serum urate and telomere length and inflammation markers: Evidence from UK Biobank cohort

**DOI:** 10.3389/fimmu.2022.1065739

**Published:** 2022-12-15

**Authors:** Zhengtao Lv, Jiarui Cui, Jiaming Zhang

**Affiliations:** ^1^ Department of Orthopedics, Tongji Hospital, Tongji Medical College, Huazhong University of Science and Technology, Wuhan, China; ^2^ School of Rehabilitation and Health Preservation, Chengdu University of Traditional Chinese Medicine, Chengdu, China

**Keywords:** serum urate, telomere length, aging, causal effect, Mendelian randomization

## Abstract

**Objective:**

Hyperuricemia and gout have become gradually more common. The effect of serum urate on organism aging and systematic inflammation is not determined. This study aims to evaluate whether serum urate is causally associated with cellular aging markers and serum inflammation markers.

**Methods:**

A Mendelian randomization study was performed on summary-level data from the largest published genome-wide association studies. Single nucleotide polymorphisms with a genome-wide significance level were selected as instrumental variables for leukocyte telomere length (LTL), and serum soluble makers of inflammation (CRP, IL-6, TNF-α, and IGF-1). Standard inverse variance weighted (IVW) method was used as the primary statistical method. The weighted median, MR-Egger regression, and MR-PRESSO methods were used for sensitivity analysis.

**Results:**

An inverse causal association of genetically predicted serum urate levels and LTL was found using IVW method (OR: 0.96, 95%CI 0.95, 0.97; β=-0.040; SE=0.0072; P=4.37×10^-8^). The association was also supported by MR results using MR-Egger method and weighted median method. The MR-PRESSO analysis and leave-one-out sensitivity analysis supported the robustness of the combined results. In terms of other aging-related serum biomarkers, there was no evidence supporting a causal effect of serum urate on CRP, IL-6, TNF-α, or IGF-1 levels.

**Conclusions:**

Serum urate levels are negatively associated with telomere length but are not associated with serum soluble indicators of inflammation. Telomere length may be a critical marker that reflects urate-related organismal aging and may be a mechanism in the age-related pathologies and mortality caused by hyperuricemia.

## Introduction

Serum urate or uric acid is an end product of purine metabolism (1). High serum urate or hyperuricemia is the most critical risk factor for gout (2). Due to the spread of Western lifestyles and diets associated with excessive meat and alcohol consumption, hyperuricemia has become gradually more common (~20.0%; US; 2016) (3). Moreover, the extensive associations of serum urate with age-related diseases, such as cardiovascular diseases (4, 5), hypertension (6), obesity (6), type 2 diabetes mellitus (7), and metabolic syndrome (8), have been implied. The effects of urate on cellular functions and signaling events, such as promoting the production of reactive oxygen species (ROS) and activating inflammatory signaling pathways, have indicated the possible involvement in systematic inflammation (9–12). Due to its complicated connections within multiple diseases, the role of urate on multiple organ systems that may be facilitated by a perturbation in circulation has been a matter of discussion. Previous studies found the associations between serum urate and serum inflammation markers, such as C-reactive protein (CRP), IL-6, and TNF-α (13). Co-occurrence of high serum urate and high CRP is associated with elevated mortality (14, 15). However, whether these associations are causal remains unclear due to potential biases such as residual confounding and reverse causality.

Telomeres are nucleoprotein structures at the ends of chromosomes that protect linear chromosomes against damage by endogenous nucleases and erroneous recognition as unrepaired chromosomal breaks (16). Due to their critical functions in the maintenance of chromosomal structure and stability (17) and the determination of cell proliferation (18), telomere length has been considered an indicator of the biological age of organisms and a biomarker of aging, stress, and survival (19–21). Telomere shortening is associated with a series of age-related deteriorations and diseases, such as cardiovascular (22) and metabolic diseases (23). Interestingly, a significant negative association between serum urate and peripheral blood aging markers, including leukocyte telomere length (LTL) and mitochondrial DNA copy number (mtDNAcn) (24), has indicated that serum urate may play a role in promoting organismal aging by telomere shortening. Although reduced telomere length and elevated serum urate levels both come with aging, a recent Mendelian randomization (MR) study does not support the casual effect of serum urate on telomere shorten (25). However, the casual association between them remains elusive because of the limited numbers of single nucleotide polymorphisms (SNPs) employed as instrumental variables (IVs) in the previous study.

Here, we used an MR design to overcome the limitations of traditional observational studies and determine whether there are causal associations between serum urate and LTL and serum soluble makers of inflammation (CRP, IL-6, TNF-α, and IGF-1) (26). To detect whether serum urate causally affects LTL, more importantly, we employed a ~4-fold larger number of SNPs as IVs than the previous MR study (25). Confounding and reverse causality can be avoided in MR analysis. This study provides reliable insight into the causal associations between serum urate and the markers of aging.

## Methods

To assess the causal association of genetically predicted urate level on aging-related biomarkers, we implemented an MR approach, assuming that (i) the SNPs used as instrumental variables (IVs) are robustly associated with urate level; (ii) the SNPs are not associated with any confounder of exposure-outcome associations; (iii) the SNPs exert effects only through urate level rather than *via* other pathways. For SNPs to instrument urate level, we obtained the results from a published meta-analysis of GWASs (27). Each GWAS in this study has been approved by relevant ethical review committees, all the participants gave written informed consent. Summary-level dataset for outcomes could be obtained from the UK Medical Research Council Integrative Epidemiology Unit (MRCIEU) Open GWAS Project database (http://gwas.mrcieu.au.uk). Therefore, no additional ethical approval is required for our study.

### Data source for urate level and instruments selection

To instrument urate level, we extracted independent SNPs significantly associated with urate level from a published European-ancestry meta-analysis of GWASs (27). The meta-analysis of urate level (n=288,649) initially yielded a total of 123 genome-wide significant SNPs (P<5.0×10^-8^) using a workflow included methods that used linkage disequilibrium (LD) estimates from an ancestry-matched reference panel (28). Then 114 independent SNPs were identified using stepwise model selection, after LD-based combination into 99 larger genomic regions. These SNPs to instrument urate levels collectively explained 11.4% of the phenotypic variance, compared to 5.3% explained by the index SNPs previously reported from GWAS in European ancestry populations (29). SNPs were removed during the harmonizing process for being palindromic with intermediate allele frequencies. The F-statistics for each SNP was calculated using the previously reported approximation method (30). SNPs with F-statistics lower than 10 were removed from our MR study to avoid weak instrument bias. Previous MR studies have suggested that several factors can causally affect serum urate level, including estimated glomerular filtration rate (eGFR) (31), type 2 diabetes (T2DM) (32), physical activity (33), body mass index (BMI) (32, 34), high-density lipoprotein cholesterol (HDL-C) (32), systolic blood pressure (SBP) (32), and triglyceride (32, 34). Any aforementioned factor that is also causally associated with our study outcomes was deemed as a confounder, and the SNPs having a causal effect on related confounders were removed from the MR analysis. After a rigorous literature search, we found that physical activity was causally associated with both serum urate level and LTL (35), but none of the selected SNPs was significantly associated with any physical activity-associated phenotype. Detailed information of selected SNPs to instrument urate level in our MR study was summarized in **Supplementary Tables S1**, **S2**.

### Data source for outcomes: LTL, CRP, IL-6, TNF-α, and IGF-1

Summary-level data (betas and SEs) for mean LTL could be obtained from a publicly available GWAS (36), which was conducted with 488,400 DNA samples of participants in the UK Biobank (UKB). Mean LTL was measured using an established quantitative PCR technique and indicated as a ratio of the telomere repeat number (T) to a single-copy gene (S) (37, 38). After extensive quality checks and adjustments of technical variations, the LTL measurements (T/S ratio) were Z-standardized to allow direct comparison with previous studies. Finally, 472,174 LTL measurements were available and retained for MR study. For serum levels of CRP, IL-6, TNF-α, and IGF-1, we extracted the summary-level data from a GWAS using 3,301 available blood samples from the INTERVAL study (39). The main characteristics of selected GWASs were presented in **Table 1**.

**Table 1:** Description of data source for each outcome.

Outcome	GWAS ID	Population	Sex	Sample size	No. of SNPs	PMID	Power at OR=0.80/1.20
LTL	ieu-b-4879	European	Males and females	472,174	20,134,421	NA	100%/100%
CRP	prot-a-670	European	Males and females	3,301	10,534,735	29875488	99%/94%
IL-6	prot-a-1538	European	Males and females	3,301	10,534,735	29875488	99%/94%
TNF-α	prot-a-3029	European	Males and females	3,301	10,534,735	29875488	99%/94%
IGF-1	prot-a-1443	European	Males and females	3,301	10,534,735	29875488	99%/94%

GWAS, genome-wide association study; SNP, single nucleotide polymorphism; LTL, leukocyte telomere length; CRP, C-reactive protein; IL-6, interleukin-6; TNF-α, tumor necrosis factor-α; IGF-1, insulin-like growth factor; NA, not available; OR, odds ratio; power was calculated assuming an OR of 0.80/1.20 per one-standard deviation increase of serum urate level.

### Statistical analysis

**Figure 1:**
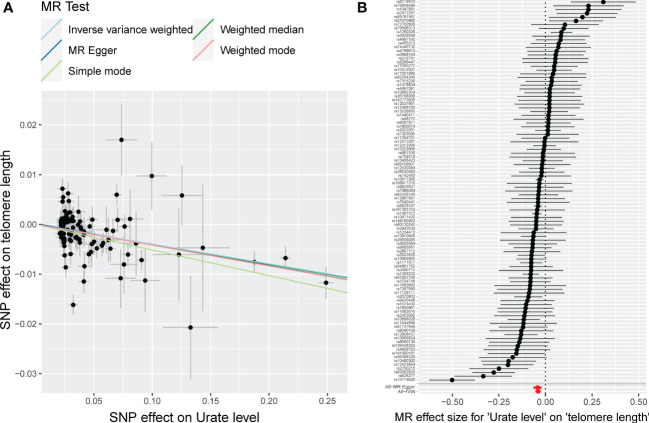
MR plots for the causal association of urate level on LTL. **(A)** Scatter plot of SNP effect on LTL against that on urate level. The slope of each fitted line represents the combined effect using each MR analysis method. **(B)** Forest plot of effect from each SNP and pooled MR effect sizes for urate level on LTL. Each dot and its corresponding line represent the effect size and 95%CI. Each dot and its corresponding line represent the pooled estimates after the removal of the corresponding SNP. LTL, leukocyte telomere length; SNP, single nucleotide polymorphism; MR, mendelian randomization.

All the analyses were performed with R (version 4.1.3), TwoSampleMR (0.5.5) (40), Mendelian Randomization (0.5.0) (41), and MR-PRESSO package (42). A scatter plot was generated to help illustrate genetic estimates (**Figure 1**). For the primary analysis, we used the standard inverse variance weighting (IVW) method under random-effect model, which assumes that each included SNP is a valid instrument (43). The IVW method combines the Wald ratios using a meta-analytic approach, but the estimates of effect could be biased by horizontal pleiotropy in the presence of invalid instruments (44). Heterogeneity between selected SNPs was evaluated using the Cochrane’s Q-statistics, significant heterogeneity could indicate the presence of horizontal pleiotropy (44).

The weighted median method, MR-Egger regression, and MR-PRESSO methods, which relax some IV assumptions were performed as sensitivity analyses. The weighted median method provides consistent estimates of causal effect when more than half of the instruments are valid (30). Confidence should be given to the weighted median method when the heterogeneity is significant and the selected SNPs are free of pleiotropy. The MR-Egger regression analysis estimates the causal effect by calculating the slope from the weighted regression of the IVs-outcome associations on the IVs-exposure associations (30). The Egger intercept indicates average pleiotropic effect, and a non-zero intercept indicates significant pleiotropy (42). In case of significant pleiotropy, we used the PhenoScanner V2 to rule out SNPs associated with potential confounders at a genome-wide significance (P<5.0×10^-8^) (45, 46). The results obtained using IVW method were deemed as the most reliable if the selected instruments are free of directional pleiotropy (P for MR-Egger intercept >0.05) (47). The MR-PRESSO approach not only detects pleiotropy by identifying outlying SNPs, but also provides outliers-corrected estimates of causal effect (42). The P value for MR-PRESSO distortion test was used to denote a significant difference in estimates before and after the correction of outlying SNPs (42). The leave-one-out sensitivity analysis was also performed to test the robustness of results. In addition, the power of our MR study was calculated according to the previously published method to test whether our study had sufficient power to determine a clinically significant association between serum urate level and study outcomes (48).

## Results

Detailed information of selected 101 SNPs to instrument urate level was summarized in **Supplementary Table 1**. The F-statistics of these selected SNPs ranged from 62.9 to 5561.5, which were much higher than the conventional threshold of 10, suggesting a low possibility of weak-instrument bias. Based on a sample size of 472,174 individuals and the SNPs explaining 10.21% of the phenotypic variance, our study had 100% power to detect an OR of 1.20 (β=-0.2231, **Table 1**). The results of our MR study suggested an inverse association between urate level and LTL. The scatter plot of the association between genetically predicted urate level and LTL was shown in **Figure 1A**, where the slope of each fitted line represented the combined effect using each MR analysis method. Using the IVW method, genetically predicted urate level was negatively associated with LTL (0.040 SD higher urate per 1-SD shorter LTL, P=4.37×10^-8^; **Table 1**). The inverse causal association was also supported by MR results using MR-Egger method and weighted median method (**Figure 1A** and **Table 2**). As shown in **Figure 1**, the MR-Egger intercept was close to zero (MR-Egger intercept=0.00018), and the MR-Egger regression analysis revealed no horizontal pleiotropy (P_-pleio_=0.76). We observed significant heterogeneity across different SNPs (P_-het_=1.34×10^-15^), thus the random-effect model was used (forest plot shown in **Figure 1B**). The MR-PRESSO approach identified four outlying SNPs, but the P value for distortion test (P=1.00) suggested that there was no significant difference between the original estimate and the outlier-corrected estimate of the causal effect. The leave-one-out sensitivity analysis also supported the robustness of the conclusion (**Supplementary Figure 1**).

**Table 2:** Causal effects of urate level on leukocyte telomere length and inflammation markers using MR analyses.

Outcome	SNPs	MR method	OR (95%CI)	Beta (SE)	P	P_-het_	P_-pleio_
LTL	101	MR Egger	0.96 (0.94, 0.98)	-0.042 (0.011)	3.46×10^-4^	1.34×10^-15^	
		MR Egger intercept		0.00018 (0.00059)			0.76
		Weighted median	0.96 (0.95, 0.97)	-0.040 (0.0075)	8.67×10^-8^		
		IVW	0.96 (0.95, 0.97)	-0.040 (0.0072)	4.37×10^-8^	2.02×10^-15^	
		MR-PRESSO distortion test			1.00		
CRP	113	MR Egger	0.87 (0.72, 1.05)	-0.014 (0.095)	0.15	0.042	
		MR Egger intercept		0.0079 (0.0049)			0.11
		Weighted median	0.88 (0.72, 1.07)	-0.13 (0.10)	0.19		
		IVW	0.98 (0.87, 1.11)	-0.017 (0.060)	0.78	0.032	
		MR-PRESSO distortion test			NA		
IL-6	113	MR Egger	0.88 (0.73, 1.06)	-0.13 (0.093)	0.17	0.098	
		MR Egger intercept		0.0060 (0.0048)			0.21
		Weighted median	0.94 (0.79, 1.11)	-0.067 (0.088)	0.45		
		IVW	0.96 (0.86, 1.08)	-0.036 (0.058)	0.53	0.091	
		MR-PRESSO distortion test			NA		
TNF-α	113	MR Egger	0.95 (0.80, 1.12)	-0.056 (0.085)	0.52	0.90	
		MR Egger intercept		0.00092 (0.0044)			0.84
		Weighted median	1.02 (0.86, 1.22)	0.023 (0.088)	0.79		
		IVW	0.96 (0.86, 1.06)	-0.042 (0.053)	0.43	0.91	
		MR-PRESSO distortion test			NA		
IGF-1	113	MR Egger	1.04 (0.87, 1.24)	0.042 (0.091)	0.65	0.17	
		MR Egger intercept		-0.0026 (0.0047)			0.58
		Weighted median	1.04 (0.87, 1.23)	0.0017 (0.056)	0.67		
		IVW	1.00 (0.90, 1.12)	0.0017 (0.056)	0.98	0.19	
		MR-PRESSO distortion test			NA		

Beta was the estimated effect size; OR, odds ratio; 95%CI, 95% confidence interval; SE, standard error; SNP, single nucleotide polymorphism; MR, mendelian randomization; LTL, leukocyte telomere length; CRP, C-reactive protein; IL-6, interleukin-6; TNF-α, tumor necrosis factor-α; IGF-1, insulin-like growth factor; IVW, inverse variance weighted; P_-het_, P value for heterogeneity test; P_-pleio_, P value for pleiotropy test using MR-Egger regression analysis; NA, not available. P<0.05 was considered statistically significant.

In terms of inflammation markers, including serum levels of CRP, IL-6, TNF-α, and IGF-1, none of our analyses suggested a causal effect of urate level (**Table 2**). Based upon a sample size of 3,301 individuals and the phenotypic variance explained to 11.24%, our study had 99%/94% power to detect an OR of 0.80/1.20 (**Table 1**).

## Discussion

Although basic research and clinical investigations have strongly indicated the implications of urate in organismal aging, its causal association with aging is not determined. In conflict with the previous MR study which reported that serum urate has no significant causal effect on LTL (25), we found that serum urate levels are reversely associated with LTL. The possible reason is that we employed a ~4-fold larger number of SNPs as IVs than the previous MR study. Moreover, the sample size of the GWAS we used in this study (27) is 4-fold larger than the previous GWAS studies (29, 49, 50). The *post hoc* power calculation suggested that our study had 100% power to detect an OR of 0.80/1.20, while the previous MR did not.

Since serum urate increases with age (51, 52), this result suggests that increased serum urate results in telomere shortening during aging. Telomere shortening is a well-documented hallmark of both cellular senescence and organismal aging, and is a critical mechanism of age-related pathologies and mortality (53). It happens in each division as a normal cellular process in the dividing cells (54). Moreover, telomere shortening occurs due to oxidative damage and other end processing events in both dividing and non-dividing cells (20). The effects of urate on cellular stress and ROS production may be the mechanism fascinating the urate-induced telomere shortening found in this study. ROS are produced simultaneously with the formation of intracellular urate by xanthine oxidases (XO) (55). Enhanced activity of XO promotes intracellular urate production. Moreover, elevated extracellular urate concentration aggravates the urate flux into cells. These events cause the accumulation of intracellular urate that aggravates ROS production (56, 57), mitochondrial damage (58), endoplasmic reticulum stress (59), and apoptosis (56). These cellular events cause telomere shortening alone or in an interactive manner; in turn, telomere shortening further aggravates cell stress, forming a closed loop (20, 60).

The role of intracellular urate has been demonstrated that it can trigger inflammation and ROS. However, we did not find a causal effect of serum urate on the markers of systematic inflammation. Previous studies have observed the associations between serum urate and serum inflammation indicators (13, 61, 62). Our study indicates that these associations are not causal. The possible interpretation of these conflict findings may be the paradox of oxidant versus anti-oxidant properties of urate in multiple systems (9) and the net effect of urate on system is variable depending on various conditions, such as genetics and disease progress.

MR studies rely on strong assumptions, and biased and misleading results could be yielded if those assumptions fail (63). In our current study, a total of 114 SNPs significantly associated with serum urate level were obtained from the largest GWAS meta-analysis, which was in line with the Relevance assumption. We then performed a comprehensive literature search to identify potential confounders that affect the exposure-outcome association. Only physical activity was deemed as a confounder through the literature search, but none of our selected SNPs was significantly associated with any physical activity-associated phenotype. It should be noted, if a particular confounder was simply not considered as a confounder in the analysis, its effect could not be accounted for. We made an effort to reduce the possibility that the Independence assumption was violated. Furthermore, we used three robust analysis methods (weighted median method, MR-Egger regression, and MR-PRESSO) as sensitivity analysis, since robust methods can provide valid causal inferences under weaker assumptions than the standard IVW method (44). Strikingly, causal estimates from all methods were similar (**Table 1**), and the concordance of estimates from different methods made the causal claim more credible.

This study has several notable strengths. First, it is the first study revealing the causal association of serum urate with telomere length and highlighting the value of telomere length as a reliable serum marker reflecting urate-related aging. Second, an integrated and comprehensive MR analysis was conducted to support the robustness of causal estimates. A series of sensitivity analyses was also employed to ensure the consistency of causal association. Third, the number of SNPs employed as IVs is relatively large, enhancing the reliability of the conclusions.

Some limitations in this study need to be considered. First, the participants involved in the study were all of European descent, which may restrict the generalizability of the findings. Second, since a linear relationship between exposure and outcome was assumed in the MR design, the potential non-linear association of serum urate with leukocyte telomere length and inflammation markers could not be evaluated. Finally, other aging-related markers, such as immune parameters and indices of epigenetic age, were not included in this study.

## Conclusions

Serum urate levels are negatively associated with telomere length but are not associated with inflammation markers. Telomere length may be a critical and reliable marker that reflects urate-related organismal aging and may be a mechanism in the age-related pathologies and mortality caused by hyperuricemia.

## Data availability statement

The original contributions presented in the study are included in the article/**Supplementary Material**. Further inquiries can be directed to the corresponding authors.

## Author contributions

ZL: conception and design, data analysis, and draft writing. JC: draft writing. JZ: draft writing, and revision. All authors approved the final manuscript.
