# Supplementary material for: Associations between serum urate and telomere length and inflammation markers: Evidence from UK Biobank cohort
Source: Front Immunol. 2022 Dec 15;13:1065739. doi: 10.3389/fimmu.2022.1065739 (PMC9797991; doi:10.3389/fimmu.2022.1065739)
Supplement: Supplementary file 1 [file DataSheet_1.docx]

**Supplementary Materials**

**Supplementary Figure 1.** The leave-one-out sensitivity analysis of the causal association of urate level on LTL. Each dot and its corresponding line represent the pooled estimates after the removal of corresponding SNP. LTL: leukocyte telomere length; SNP: single nucleotide polymorphism; MR: mendelian randomization.

**Supplementary Table 1.** Summary statistics of SNPs for urate level to estimate the causal effect on LTL.

**Supplementary Table 2.** Summary statistics of SNPs for urate level to estimate the causal effect on CRP, IL-6, TNF-α, and IGF-1.

**Supplementary Figure 1. The leave-one-out sensitivity analysis of the causal association of urate level on LTL.**


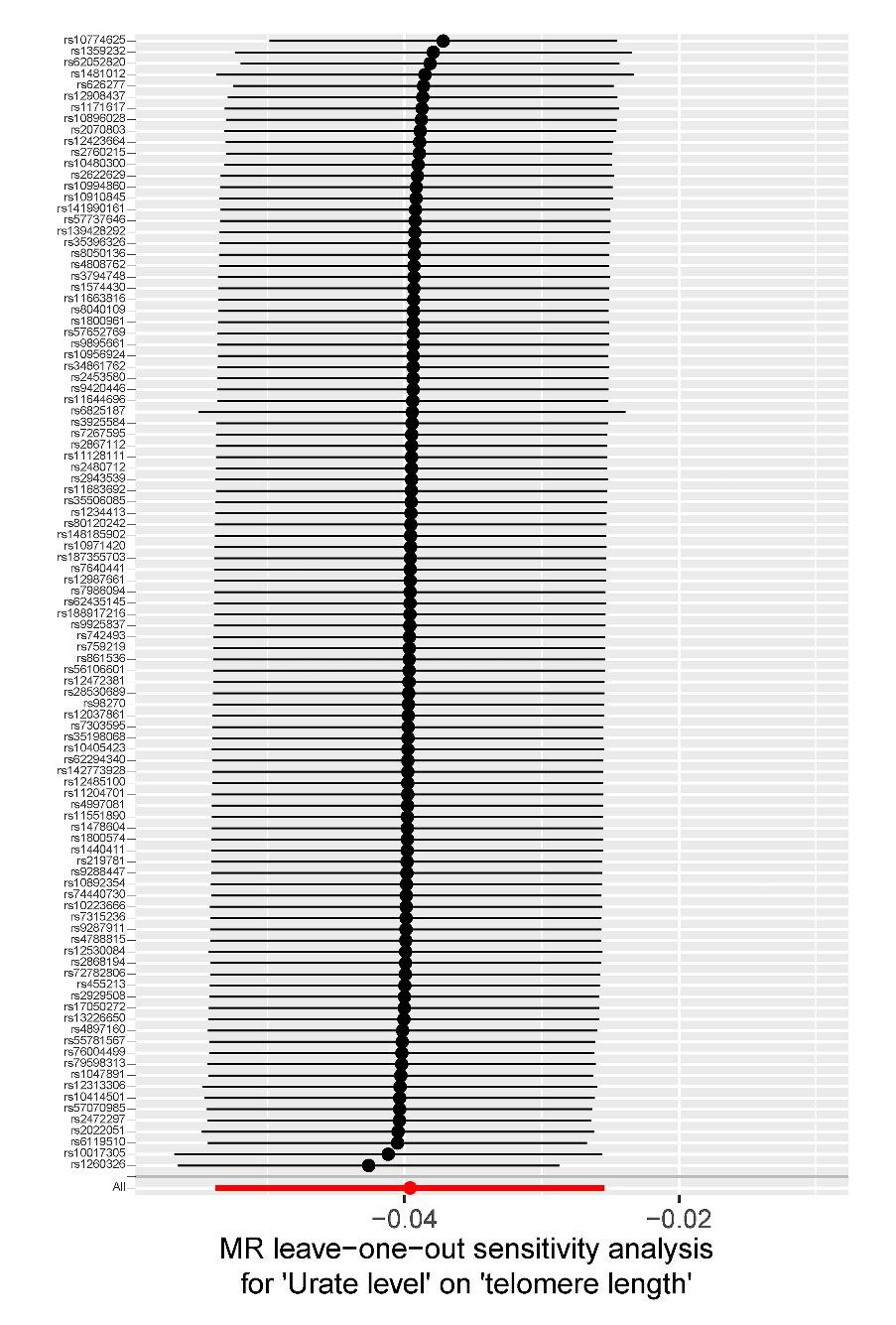


Each dot and its corresponding line represent the pooled estimates after the removal of corresponding SNP. LTL: leukocyte telomere length; SNP: single nucleotide polymorphism; MR: mendelian randomization.

| **Supplementary Table 1. Summary statistics of SNPs for urate level to estimate the causal effect on LTL.** | | | | | | | | | | | | |
| --- | --- | --- | --- | --- | --- | --- | --- | --- | --- | --- | --- | --- |
| **SNP** | **Chr** | **Position** | **EA** | **OA** | **EAF** | **Beta** | **SE** | **P value** | **Nearest gene** | **R^2^** | **F** |  |
| rs10910845 | 1 | 145723120 | A | C | 0.4692 | 0.057964 | 0.0038374 | 1.5E-51 | NBPF20 | 0.0016735 | 483.872 |  |
| rs11204701 | 1 | 150662179 | A | T | 0.7788 | -0.03639 | 0.0047054 | 1.05E-14 | GOLPH3L | 0.0004563 | 131.756 |  |
| rs12037861 | 1 | 221038177 | A | T | 0.7041 | 0.023078 | 0.0041809 | 3.39E-08 | HLX-AS1 | 0.0002219 | 64.072 |  |
| rs139428292 | 1 | 145507646 | A | G | 0.0266 | -0.072898 | 0.0133646 | 4.91E-08 | NBPF20 | 0.0002752 | 79.455 |  |
| rs141990161 | 1 | 119943525 | T | C | 0.9849 | 0.132789 | 0.0234958 | 1.59E-08 | HAO2 | 0.0005245 | 151.467 |  |
| rs2070803 | 1 | 155157715 | A | G | 0.5777 | 0.052586 | 0.0039158 | 4.09E-41 | TRIM46 | 0.0013493 | 389.984 |  |
| rs2480712 | 1 | 2156999 | C | G | 0.6621 | 0.024325 | 0.0041868 | 6.25E-09 | SKI | 0.0002648 | 76.442 |  |
| rs2760215 | 1 | 163675883 | T | C | 0.5034 | -0.024985 | 0.0038153 | 5.81E-11 | LOC100422212 | 0.0003121 | 90.118 |  |
| rs79598313 | 1 | 27284913 | T | C | 0.0260 | 0.09962 | 0.0128549 | 9.22E-15 | KDF1 | 0.0005026 | 145.158 |  |
| rs1047891 | 2 | 211540507 | A | C | 0.3107 | -0.023735 | 0.0042351 | 2.09E-08 | CPS1 | 0.0002413 | 69.667 |  |
| rs11683692 | 2 | 145509615 | T | C | 0.9444 | -0.048213 | 0.0084831 | 1.32E-08 | TEX41 | 0.0002441 | 70.479 |  |
| rs1234413 | 2 | 148844369 | T | C | 0.4415 | -0.022345 | 0.0038599 | 7.08E-09 | MBD5 | 0.0002462 | 71.092 |  |
| rs12472381 | 2 | 59321225 | A | G | 0.3900 | 0.021931 | 0.0038952 | 1.8E-08 | LINC01122 | 0.0002288 | 66.071 |  |
| rs1260326 | 2 | 27730940 | T | C | 0.3983 | 0.069602 | 0.0039626 | 4.61E-69 | GCKR | 0.002322 | 671.801 |  |
| rs12987661 | 2 | 69813458 | T | C | 0.8657 | 0.041324 | 0.0058364 | 1.44E-12 | AAK1 | 0.0003971 | 114.662 |  |
| rs17050272 | 2 | 121306440 | A | G | 0.4210 | 0.031806 | 0.00399 | 1.57E-15 | LINC01101 | 0.0004932 | 142.426 |  |
| rs187355703 | 2 | 176993583 | C | G | 0.9747 | -0.086139 | 0.0129299 | 2.7E-11 | HOXD8 | 0.0003659 | 105.669 |  |
| rs2867112 | 2 | 651349 | T | G | 0.8301 | 0.035023 | 0.0051437 | 9.84E-12 | TMEM18 | 0.000346 | 99.903 |  |
| rs72782806 | 2 | 15788511 | A | G | 0.2595 | 0.025282 | 0.0043848 | 8.12E-09 | DDX1 | 0.0002456 | 70.923 |  |
| rs759219 | 2 | 71163225 | T | C | 0.4387 | -0.022269 | 0.0038595 | 7.93E-09 | ATP6V1B1 | 0.0002442 | 70.513 |  |
| rs9287911 | 2 | 170037294 | A | T | 0.2497 | 0.038206 | 0.0044632 | 1.13E-17 | LRP2 | 0.0005469 | 157.962 |  |
| rs9288447 | 2 | 213083638 | T | C | 0.5458 | -0.022527 | 0.0038068 | 3.27E-09 | ERBB4 | 0.0002516 | 72.643 |  |
| rs11128111 | 3 | 69145632 | T | C | 0.4800 | -0.020891 | 0.003823 | 4.64E-08 | ARL6IP5 | 0.0002179 | 62.901 |  |
| rs62294340 | 3 | 169155476 | A | G | 0.3640 | -0.021858 | 0.0040096 | 5E-08 | MECOM | 0.0002212 | 63.866 |  |
| rs7640441 | 3 | 125118082 | A | C | 0.2457 | -0.027525 | 0.0045325 | 1.26E-09 | ZNF148 | 0.0002808 | 81.082 |  |
| rs80120242 | 3 | 132235344 | A | T | 0.9467 | -0.062418 | 0.0103875 | 1.87E-09 | DNAJC13 | 0.0003932 | 113.534 |  |
| rs10017305 | 4 | 10401223 | T | C | 0.7092 | 0.214079 | 0.0045559 | 0 | ZNF518B | 0.0189035 | 5561.559 |  |
| rs1440411 | 4 | 144158285 | T | C | 0.5706 | -0.027552 | 0.0038698 | 1.08E-12 | USP38 | 0.000372 | 107.413 |  |
| rs1481012 | 4 | 89039082 | A | G | 0.8893 | -0.248838 | 0.006194 | 0 | ABCG2 | 0.0121916 | 3562.490 |  |
| rs188917216 | 4 | 88872920 | A | C | 0.9883 | -0.14329 | 0.0231886 | 6.44E-10 | SPP1 | 0.0004748 | 137.123 |  |
| rs2622629 | 4 | 89094064 | T | C | 0.6359 | -0.056927 | 0.0041015 | 8.42E-44 | ABCG2 | 0.0015006 | 433.806 |  |
| rs6825187 | 4 | 9915325 | T | C | 0.3573 | 0.187461 | 0.0040172 | 0 | SLC2A9 | 0.0161396 | 4735.074 |  |
| rs98270 | 4 | 48019323 | A | G | 0.3620 | 0.021718 | 0.0039618 | 4.21E-08 | NIPAL1 | 0.0002179 | 62.901 |  |
| rs455213 | 5 | 34660235 | T | C | 0.5428 | -0.026532 | 0.0038574 | 6.05E-12 | RAI14 | 0.0003494 | 100.887 |  |
| rs76004499 | 5 | 176705865 | C | G | 0.9722 | -0.073671 | 0.0133303 | 3.27E-08 | NSD1 | 0.0002934 | 84.707 |  |
| rs10223666 | 6 | 43805502 | C | G | 0.7036 | 0.046445 | 0.0042414 | 6.62E-28 | VEGFA | 0.0008997 | 259.938 |  |
| rs12530084 | 6 | 7214676 | T | C | 0.2198 | 0.06643 | 0.0045762 | 9.55E-48 | RREB1 | 0.0015135 | 437.539 |  |
| rs1359232 | 6 | 25809716 | A | C | 0.4660 | -0.090844 | 0.003803 | 4.1E-126 | SLC17A1 | 0.0041072 | 1190.431 |  |
| rs1574430 | 6 | 43269029 | A | C | 0.4051 | 0.029394 | 0.0038633 | 2.77E-14 | SLC22A7 | 0.0004164 | 120.255 |  |
| rs4897160 | 6 | 126223944 | A | G | 0.4828 | 0.029735 | 0.0038854 | 1.96E-14 | NCOA7 | 0.0004416 | 127.512 |  |
| rs742493 | 6 | 40998167 | T | C | 0.8808 | 0.039012 | 0.0060295 | 9.79E-11 | UNC5CL | 0.0003196 | 92.275 |  |
| rs10480300 | 7 | 151406005 | T | C | 0.2755 | 0.030123 | 0.0043479 | 4.26E-12 | PRKAG2 | 0.0003622 | 104.595 |  |
| rs11551890 | 7 | 97845713 | A | G | 0.5087 | 0.02308 | 0.0041358 | 2.4E-08 | TECPR1 | 0.0002663 | 76.876 |  |
| rs13226650 | 7 | 73017005 | A | G | 0.8085 | 0.048744 | 0.0048683 | 1.35E-23 | MLXIPL | 0.0007357 | 212.524 |  |
| rs62435145 | 7 | 1286567 | T | G | 0.6891 | 0.041662 | 0.0050793 | 2.36E-16 | UNCX | 0.0007437 | 214.834 |  |
| rs10956924 | 8 | 95678312 | T | C | 0.2793 | -0.023868 | 0.0042382 | 1.79E-08 | ESRP1 | 0.0002293 | 66.215 |  |
| rs2943539 | 8 | 76479839 | T | C | 0.4754 | 0.04144 | 0.0037834 | 6.42E-28 | HNF4G | 0.0008566 | 247.455 |  |
| rs34861762 | 8 | 23748420 | T | C | 0.4194 | 0.034282 | 0.0038297 | 3.5E-19 | STC1 | 0.0005724 | 165.304 |  |
| rs10971420 | 9 | 33125000 | T | C | 0.6875 | 0.030745 | 0.0040688 | 4.14E-14 | B4GALT1 | 0.0004062 | 117.286 |  |
| rs56106601 | 9 | 130770484 | A | C | 0.9455 | 0.060781 | 0.0091216 | 2.68E-11 | FAM102A | 0.0003807 | 109.940 |  |
| rs10994860 | 10 | 52645424 | T | C | 0.1801 | 0.064207 | 0.0050806 | 1.31E-36 | A1CF | 0.0012175 | 351.856 |  |
| rs1171617 | 10 | 61467182 | T | G | 0.7682 | 0.079207 | 0.0045991 | 1.81E-66 | SLC16A9 | 0.0022343 | 646.373 |  |
| rs35198068 | 10 | 114754784 | T | C | 0.7063 | 0.024734 | 0.0042491 | 5.85E-09 | TCF7L2 | 0.0002538 | 73.281 |  |
| rs74440730 | 10 | 16920892 | A | C | 0.8924 | -0.03682 | 0.0061563 | 2.22E-09 | CUBN | 0.0002604 | 75.171 |  |
| rs9420446 | 10 | 88880689 | T | C | 0.1371 | -0.038016 | 0.0055996 | 1.13E-11 | FAM35A | 0.0003419 | 98.736 |  |
| rs10892354 | 11 | 119238381 | T | C | 0.3800 | 0.030101 | 0.0041158 | 2.6E-13 | USP2 | 0.0004269 | 123.288 |  |
| rs10896028 | 11 | 65432187 | A | T | 0.6451 | -0.047581 | 0.003969 | 4.1E-33 | RELA | 0.0010366 | 299.535 |  |
| rs148185902 | 11 | 30718534 | A | G | 0.0116 | 0.122789 | 0.0226295 | 5.76E-08 | MPPED2 | 0.0003457 | 99.829 |  |
| rs2022051 | 11 | 64367589 | A | G | 0.7934 | -0.070369 | 0.0048334 | 5.13E-48 | SLC22A12 | 0.0016234 | 469.340 |  |
| rs35506085 | 11 | 2165576 | A | G | 0.1887 | -0.028758 | 0.005079 | 1.5E-08 | IGF2 | 0.0002532 | 73.110 |  |
| rs3925584 | 11 | 30760335 | T | C | 0.5522 | 0.030389 | 0.0038157 | 1.66E-15 | DCDC1 | 0.0004567 | 131.889 |  |
| rs10774625 | 12 | 111910219 | A | G | 0.4826 | 0.032335 | 0.003861 | 5.54E-17 | ATXN2 | 0.0005221 | 150.794 |  |
| rs12313306 | 12 | 57751854 | T | C | 0.2464 | -0.076333 | 0.0044871 | 6.74E-65 | R3HDM2 | 0.0021639 | 625.956 |  |
| rs12423664 | 12 | 133069894 | A | G | 0.1515 | 0.041927 | 0.0056912 | 1.75E-13 | FBRSL1 | 0.0004519 | 130.510 |  |
| rs1800574 | 12 | 121416864 | T | C | 0.0314 | -0.080982 | 0.011593 | 2.84E-12 | HNF1A | 0.0003989 | 115.192 |  |
| rs28530689 | 12 | 122500748 | A | C | 0.5117 | 0.032213 | 0.0038922 | 1.27E-16 | LOC100506691 | 0.0005186 | 149.757 |  |
| rs7303595 | 12 | 15359063 | A | T | 0.3357 | 0.025313 | 0.004106 | 7.05E-10 | RERG | 0.0002858 | 82.513 |  |
| rs7315236 | 12 | 52251933 | T | C | 0.3570 | 0.029135 | 0.0039611 | 1.91E-13 | LOC105369971 | 0.0003897 | 112.532 |  |
| rs626277 | 13 | 72347696 | A | C | 0.5938 | 0.025914 | 0.0038894 | 2.69E-11 | DACH1 | 0.000324 | 93.538 |  |
| rs7986094 | 13 | 31029931 | A | C | 0.3016 | -0.023916 | 0.0042434 | 1.74E-08 | HMGB1 | 0.000241 | 69.569 |  |
| rs861536 | 14 | 104167564 | A | G | 0.6210 | 0.0238 | 0.0039764 | 2.16E-09 | KLC1 | 0.0002666 | 76.983 |  |
| rs12908437 | 15 | 99287375 | T | C | 0.3761 | 0.045764 | 0.0039845 | 1.56E-30 | IGF1R | 0.0009829 | 283.982 |  |
| rs1478604 | 15 | 39873321 | T | C | 0.7061 | -0.026249 | 0.0042092 | 4.49E-10 | THBS1 | 0.000286 | 82.568 |  |
| rs2472297 | 15 | 75027880 | T | C | 0.2487 | -0.027944 | 0.0049352 | 1.5E-08 | CYP1A1 | 0.0002918 | 84.254 |  |
| rs2929508 | 15 | 72246964 | A | T | 0.2608 | -0.028981 | 0.0049126 | 3.65E-09 | MYO9A | 0.0003238 | 93.505 |  |
| rs55781567 | 15 | 78857986 | C | G | 0.6548 | 0.023147 | 0.0040512 | 1.11E-08 | CHRNA5 | 0.0002422 | 69.931 |  |
| rs57737646 | 15 | 76299828 | T | C | 0.0252 | -0.093941 | 0.012489 | 5.4E-14 | NRG4 | 0.0004336 | 125.202 |  |
| rs8040109 | 15 | 73334225 | A | C | 0.7074 | 0.024914 | 0.00428 | 5.85E-09 | NEO1 | 0.000257 | 74.188 |  |
| rs11644696 | 16 | 81572093 | A | G | 0.4766 | 0.022064 | 0.0038899 | 1.41E-08 | CMIP | 0.0002429 | 70.123 |  |
| rs4788815 | 16 | 71634811 | A | T | 0.3570 | -0.026241 | 0.00403 | 7.44E-11 | TAT | 0.0003161 | 91.280 |  |
| rs4997081 | 16 | 20365234 | C | G | 0.1962 | -0.030192 | 0.0048328 | 4.18E-10 | UMOD | 0.0002875 | 83.014 |  |
| rs57652769 | 16 | 79753976 | T | C | 0.3094 | -0.036197 | 0.0042129 | 8.56E-18 | MAFTRR | 0.0005599 | 161.708 |  |
| rs62052820 | 16 | 69575238 | A | G | 0.2124 | 0.041401 | 0.0047484 | 2.81E-18 | MIR1538 | 0.0005735 | 165.626 |  |
| rs8050136 | 16 | 53816275 | A | C | 0.4029 | 0.02464 | 0.0038882 | 2.34E-10 | FTO | 0.0002921 | 84.343 |  |
| rs9925837 | 16 | 79927303 | A | G | 0.8445 | -0.041628 | 0.0053322 | 5.85E-15 | LINC01229 | 0.0004551 | 131.430 |  |
| rs2453580 | 17 | 19438321 | T | C | 0.5977 | 0.024703 | 0.0040064 | 7.01E-10 | SLC47A1 | 0.0002935 | 84.734 |  |
| rs3794748 | 17 | 53365172 | A | G | 0.4088 | 0.037619 | 0.0039418 | 1.38E-21 | HLF | 0.0006841 | 197.585 |  |
| rs9895661 | 17 | 59456589 | T | C | 0.8174 | 0.050208 | 0.0051 | 7.23E-23 | BCAS3 | 0.0007525 | 217.373 |  |
| rs11663816 | 18 | 57876227 | T | C | 0.7295 | -0.030377 | 0.0042881 | 1.4E-12 | MC4R | 0.0003642 | 105.157 |  |
| rs10405423 | 19 | 7211311 | A | C | 0.6625 | 0.03865 | 0.004143 | 1.07E-20 | INSR | 0.000668 | 192.951 |  |
| rs10414501 | 19 | 50259674 | C | G | 0.9570 | -0.125438 | 0.0177451 | 1.56E-12 | TSKS | 0.001295 | 374.282 |  |
| rs2868194 | 19 | 33350060 | T | C | 0.4084 | -0.026759 | 0.0039217 | 8.9E-12 | SLC7A9 | 0.000346 | 99.908 |  |
| rs35396326 | 19 | 45357003 | C | G | 0.7040 | 0.024742 | 0.0044281 | 2.31E-08 | NECTIN2 | 0.0002551 | 73.662 |  |
| rs4808762 | 19 | 18326222 | T | C | 0.7199 | -0.024229 | 0.0042668 | 1.36E-08 | PDE4C | 0.0002367 | 68.353 |  |
| rs57070985 | 19 | 4969053 | A | G | 0.6461 | 0.028568 | 0.0040629 | 2.04E-12 | KDM4B | 0.0003732 | 107.770 |  |
| rs142773928 | 20 | 43038720 | A | G | 0.1660 | 0.031688 | 0.0053763 | 3.77E-09 | HNF4A | 0.000278 | 80.275 |  |
| rs1800961 | 20 | 43042364 | T | C | 0.0339 | -0.075792 | 0.0118559 | 1.63E-10 | HNF4A | 0.0003763 | 108.650 |  |
| rs6119510 | 20 | 33287782 | T | G | 0.5959 | -0.023095 | 0.0039005 | 3.2E-09 | TP53INP2 | 0.0002569 | 74.166 |  |
| rs7267595 | 20 | 10643850 | A | C | 0.5097 | 0.022524 | 0.0038025 | 3.15E-09 | JAG1 | 0.0002536 | 73.211 |  |
| rs219781 | 21 | 37832621 | T | G | 0.2456 | -0.025151 | 0.0044479 | 1.56E-08 | CLDN14 | 0.0002344 | 67.677 |  |
| rs12485100 | 22 | 44325516 | T | G | 0.1726 | -0.03262 | 0.0051526 | 2.44E-10 | PNPLA3 | 0.0003039 | 87.751 |  |

| **Supplementary Table 2. Summary statistics of SNPs for urate level to estimate the causal effect on CRP, IL-6, TNF-α, and IGF-1.** | | | | | | | | | | | |
| --- | --- | --- | --- | --- | --- | --- | --- | --- | --- | --- | --- |
| **SNP** | **Chr** | **Position** | **EA** | **OA** | **EAF** | **Beta** | **SE** | **P value** | **Nearest gene** | **R^2^** | **F** |
| rs10910845 | 1 | 145723120 | A | C | 0.4692 | 0.057964 | 0.0038374 | 1.5E-51 | NBPF20 | 0.0016735 | 483.872 |
| rs11204701 | 1 | 150662179 | A | T | 0.7788 | -0.03639 | 0.0047054 | 1.05E-14 | GOLPH3L | 0.0004563 | 131.756 |
| rs12037861 | 1 | 221038177 | A | T | 0.7041 | 0.023078 | 0.0041809 | 3.39E-08 | HLX-AS1 | 0.0002219 | 64.072 |
| rs139428292 | 1 | 145507646 | A | G | 0.0266 | -0.072898 | 0.0133646 | 4.91E-08 | NBPF20 | 0.0002752 | 79.455 |
| rs141990161 | 1 | 119943525 | T | C | 0.9849 | 0.132789 | 0.0234958 | 1.59E-08 | HAO2 | 0.0005245 | 151.467 |
| rs2070803 | 1 | 155157715 | A | G | 0.5777 | 0.052586 | 0.0039158 | 4.09E-41 | TRIM46 | 0.0013493 | 389.984 |
| rs2480712 | 1 | 2156999 | C | G | 0.6621 | 0.024325 | 0.0041868 | 6.25E-09 | SKI | 0.0002648 | 76.442 |
| rs2760215 | 1 | 163675883 | T | C | 0.5034 | -0.024985 | 0.0038153 | 5.81E-11 | LOC100422212 | 0.0003121 | 90.118 |
| rs4646068 | 1 | 15828704 | T | C | 0.6920 | 0.02366 | 0.0040972 | 7.71E-09 | CASP9 | 0.0002386 | 68.895 |
| rs79598313 | 1 | 27284913 | T | C | 0.0260 | 0.09962 | 0.0128549 | 9.22E-15 | KDF1 | 0.0005026 | 145.158 |
| rs1047891 | 2 | 211540507 | A | C | 0.3107 | -0.023735 | 0.0042351 | 2.09E-08 | CPS1 | 0.0002413 | 69.667 |
| rs11683692 | 2 | 145509615 | T | C | 0.9444 | -0.048213 | 0.0084831 | 1.32E-08 | TEX41 | 0.0002441 | 70.479 |
| rs1234413 | 2 | 148844369 | T | C | 0.4415 | -0.022345 | 0.0038599 | 7.08E-09 | MBD5 | 0.0002462 | 71.092 |
| rs12472381 | 2 | 59321225 | A | G | 0.3900 | 0.021931 | 0.0038952 | 1.8E-08 | LINC01122 | 0.0002288 | 66.071 |
| rs1260326 | 2 | 27730940 | T | C | 0.3983 | 0.069602 | 0.0039626 | 4.61E-69 | GCKR | 0.002322 | 671.801 |
| rs12987661 | 2 | 69813458 | T | C | 0.8657 | 0.041324 | 0.0058364 | 1.44E-12 | AAK1 | 0.0003971 | 114.662 |
| rs17050272 | 2 | 121306440 | A | G | 0.4210 | 0.031806 | 0.00399 | 1.57E-15 | LINC01101 | 0.0004932 | 142.426 |
| rs187355703 | 2 | 176993583 | C | G | 0.9747 | -0.086139 | 0.0129299 | 2.7E-11 | HOXD8 | 0.0003659 | 105.669 |
| rs2867112 | 2 | 651349 | T | G | 0.8301 | 0.035023 | 0.0051437 | 9.84E-12 | TMEM18 | 0.000346 | 99.903 |
| rs72782806 | 2 | 15788511 | A | G | 0.2595 | 0.025282 | 0.0043848 | 8.12E-09 | DDX1 | 0.0002456 | 70.923 |
| rs759219 | 2 | 71163225 | T | C | 0.4387 | -0.022269 | 0.0038595 | 7.93E-09 | ATP6V1B1 | 0.0002442 | 70.513 |
| rs9287911 | 2 | 170037294 | A | T | 0.2497 | 0.038206 | 0.0044632 | 1.13E-17 | LRP2 | 0.0005469 | 157.962 |
| rs9288447 | 2 | 213083638 | T | C | 0.5458 | -0.022527 | 0.0038068 | 3.27E-09 | ERBB4 | 0.0002516 | 72.643 |
| rs11128111 | 3 | 69145632 | T | C | 0.4800 | -0.020891 | 0.003823 | 4.64E-08 | ARL6IP5 | 0.0002179 | 62.901 |
| rs11718633 | 3 | 126012421 | T | C | 0.1980 | -0.027933 | 0.0048246 | 7.05E-09 | KLF15 | 0.0002478 | 71.545 |
| rs62294340 | 3 | 169155476 | A | G | 0.3640 | -0.021858 | 0.0040096 | 5E-08 | MECOM | 0.0002212 | 63.866 |
| rs7640441 | 3 | 125118082 | A | C | 0.2457 | -0.027525 | 0.0045325 | 1.26E-09 | ZNF148 | 0.0002808 | 81.082 |
| rs80120242 | 3 | 132235344 | A | T | 0.9467 | -0.062418 | 0.0103875 | 1.87E-09 | DNAJC13 | 0.0003932 | 113.534 |
| rs10017305 | 4 | 10401223 | T | C | 0.7092 | 0.214079 | 0.0045559 | 0 | ZNF518B | 0.0189035 | 5561.559 |
| rs10857147 | 4 | 81181072 | A | T | 0.7130 | 0.024332 | 0.0043494 | 2.21E-08 | FGF5 | 0.0002423 | 69.957 |
| rs1440411 | 4 | 144158285 | T | C | 0.5706 | -0.027552 | 0.0038698 | 1.08E-12 | USP38 | 0.000372 | 107.413 |
| rs1481012 | 4 | 89039082 | A | G | 0.8893 | -0.248838 | 0.006194 | 0 | ABCG2 | 0.0121916 | 3562.490 |
| rs188917216 | 4 | 88872920 | A | C | 0.9883 | -0.14329 | 0.0231886 | 6.44E-10 | SPP1 | 0.0004748 | 137.123 |
| rs2622629 | 4 | 89094064 | T | C | 0.6359 | -0.056927 | 0.0041015 | 8.42E-44 | ABCG2 | 0.0015006 | 433.806 |
| rs62286563 | 4 | 10122665 | T | G | 0.9792 | -0.103135 | 0.0145057 | 1.16E-12 | WDR1 | 0.0004333 | 125.122 |
| rs6825187 | 4 | 9915325 | T | C | 0.3573 | 0.187461 | 0.0040172 | 0 | SLC2A9 | 0.0161396 | 4735.074 |
| rs73224492 | 4 | 10440925 | A | G | 0.8745 | -0.094051 | 0.0058184 | 9E-59 | ZNF518B | 0.0019416 | 561.528 |
| rs98270 | 4 | 48019323 | A | G | 0.3620 | 0.021718 | 0.0039618 | 4.21E-08 | NIPAL1 | 0.0002179 | 62.901 |
| rs10942549 | 5 | 72426137 | C | G | 0.3120 | -0.042149 | 0.0043177 | 1.64E-22 | TMEM171 | 0.0007627 | 220.316 |
| rs455213 | 5 | 34660235 | T | C | 0.5428 | -0.026532 | 0.0038574 | 6.05E-12 | RAI14 | 0.0003494 | 100.887 |
| rs76004499 | 5 | 176705865 | C | G | 0.9722 | -0.073671 | 0.0133303 | 3.27E-08 | NSD1 | 0.0002934 | 84.707 |
| rs10223666 | 6 | 43805502 | C | G | 0.7036 | 0.046445 | 0.0042414 | 6.62E-28 | VEGFA | 0.0008997 | 259.938 |
| rs12530084 | 6 | 7214676 | T | C | 0.2198 | 0.06643 | 0.0045762 | 9.55E-48 | RREB1 | 0.0015135 | 437.539 |
| rs1359232 | 6 | 25809716 | A | C | 0.4660 | -0.090844 | 0.003803 | 4.1E-126 | SLC17A1 | 0.0041072 | 1190.431 |
| rs1574430 | 6 | 43269029 | A | C | 0.4051 | 0.029394 | 0.0038633 | 2.77E-14 | SLC22A7 | 0.0004164 | 120.255 |
| rs198851 | 6 | 26104632 | T | G | 0.1440 | 0.038954 | 0.0054064 | 5.8E-13 | HIST1H4C | 0.0003741 | 108.019 |
| rs4897160 | 6 | 126223944 | A | G | 0.4828 | 0.029735 | 0.0038854 | 1.96E-14 | NCOA7 | 0.0004416 | 127.512 |
| rs742493 | 6 | 40998167 | T | C | 0.8808 | 0.039012 | 0.0060295 | 9.79E-11 | UNC5CL | 0.0003196 | 92.275 |
| rs10480300 | 7 | 151406005 | T | C | 0.2755 | 0.030123 | 0.0043479 | 4.26E-12 | PRKAG2 | 0.0003622 | 104.595 |
| rs11551890 | 7 | 97845713 | A | G | 0.5087 | 0.02308 | 0.0041358 | 2.4E-08 | TECPR1 | 0.0002663 | 76.876 |
| rs13226650 | 7 | 73017005 | A | G | 0.8085 | 0.048744 | 0.0048683 | 1.35E-23 | MLXIPL | 0.0007357 | 212.524 |
| rs62435145 | 7 | 1286567 | T | G | 0.6891 | 0.041662 | 0.0050793 | 2.36E-16 | UNCX | 0.0007437 | 214.834 |
| rs10956924 | 8 | 95678312 | T | C | 0.2793 | -0.023868 | 0.0042382 | 1.79E-08 | ESRP1 | 0.0002293 | 66.215 |
| rs2466077 | 8 | 32432753 | T | G | 0.5332 | -0.021748 | 0.0038617 | 1.78E-08 | NRG1 | 0.0002354 | 67.977 |
| rs2943539 | 8 | 76479839 | T | C | 0.4754 | 0.04144 | 0.0037834 | 6.42E-28 | HNF4G | 0.0008566 | 247.455 |
| rs34861762 | 8 | 23748420 | T | C | 0.4194 | 0.034282 | 0.0038297 | 3.5E-19 | STC1 | 0.0005724 | 165.304 |
| rs10971420 | 9 | 33125000 | T | C | 0.6875 | 0.030745 | 0.0040688 | 4.14E-14 | B4GALT1 | 0.0004062 | 117.286 |
| rs56106601 | 9 | 130770484 | A | C | 0.9455 | 0.060781 | 0.0091216 | 2.68E-11 | FAM102A | 0.0003807 | 109.940 |
| rs10994860 | 10 | 52645424 | T | C | 0.1801 | 0.064207 | 0.0050806 | 1.31E-36 | A1CF | 0.0012175 | 351.856 |
| rs1171617 | 10 | 61467182 | T | G | 0.7682 | 0.079207 | 0.0045991 | 1.81E-66 | SLC16A9 | 0.0022343 | 646.373 |
| rs1649078 | 10 | 60293320 | A | C | 0.4796 | -0.03863 | 0.0043236 | 4.09E-19 | BICC1 | 0.0007449 | 215.172 |
| rs35198068 | 10 | 114754784 | T | C | 0.7063 | 0.024734 | 0.0042491 | 5.85E-09 | TCF7L2 | 0.0002538 | 73.281 |
| rs74440730 | 10 | 16920892 | A | C | 0.8924 | -0.03682 | 0.0061563 | 2.22E-09 | CUBN | 0.0002604 | 75.171 |
| rs9420446 | 10 | 88880689 | T | C | 0.1371 | -0.038016 | 0.0055996 | 1.13E-11 | FAM35A | 0.0003419 | 98.736 |
| rs10892354 | 11 | 119238381 | T | C | 0.3800 | 0.030101 | 0.0041158 | 2.6E-13 | USP2 | 0.0004269 | 123.288 |
| rs10896028 | 11 | 65432187 | A | T | 0.6451 | -0.047581 | 0.003969 | 4.1E-33 | RELA | 0.0010366 | 299.535 |
| rs148185902 | 11 | 30718534 | A | G | 0.0116 | 0.122789 | 0.0226295 | 5.76E-08 | MPPED2 | 0.0003457 | 99.829 |
| rs2022051 | 11 | 64367589 | A | G | 0.7934 | -0.070369 | 0.0048334 | 5.13E-48 | SLC22A12 | 0.0016234 | 469.340 |
| rs35506085 | 11 | 2165576 | A | G | 0.1887 | -0.028758 | 0.005079 | 1.5E-08 | IGF2 | 0.0002532 | 73.110 |
| rs3925584 | 11 | 30760335 | T | C | 0.5522 | 0.030389 | 0.0038157 | 1.66E-15 | DCDC1 | 0.0004567 | 131.889 |
| rs71456318 | 11 | 64332862 | A | C | 0.4842 | 0.079027 | 0.0038829 | 4.41E-92 | SLC22A11 | 0.0031195 | 903.256 |
| rs10774625 | 12 | 111910219 | A | G | 0.4826 | 0.032335 | 0.003861 | 5.54E-17 | ATXN2 | 0.0005221 | 150.794 |
| rs12313306 | 12 | 57751854 | T | C | 0.2464 | -0.076333 | 0.0044871 | 6.74E-65 | R3HDM2 | 0.0021639 | 625.956 |
| rs12423664 | 12 | 133069894 | A | G | 0.1515 | 0.041927 | 0.0056912 | 1.75E-13 | FBRSL1 | 0.0004519 | 130.510 |
| rs1800574 | 12 | 121416864 | T | C | 0.0314 | -0.080982 | 0.011593 | 2.84E-12 | HNF1A | 0.0003989 | 115.192 |
| rs28530689 | 12 | 122500748 | A | C | 0.5117 | 0.032213 | 0.0038922 | 1.27E-16 | LOC100506691 | 0.0005186 | 149.757 |
| rs7303595 | 12 | 15359063 | A | T | 0.3357 | 0.025313 | 0.004106 | 7.05E-10 | RERG | 0.0002858 | 82.513 |
| rs7315236 | 12 | 52251933 | T | C | 0.3570 | 0.029135 | 0.0039611 | 1.91E-13 | LOC105369971 | 0.0003897 | 112.532 |
| rs626277 | 13 | 72347696 | A | C | 0.5938 | 0.025914 | 0.0038894 | 2.69E-11 | DACH1 | 0.000324 | 93.538 |
| rs7986094 | 13 | 31029931 | A | C | 0.3016 | -0.023916 | 0.0042434 | 1.74E-08 | HMGB1 | 0.000241 | 69.569 |
| rs861536 | 14 | 104167564 | A | G | 0.6210 | 0.0238 | 0.0039764 | 2.16E-09 | KLC1 | 0.0002666 | 76.983 |
| rs10851885 | 15 | 76304503 | A | G | 0.7559 | -0.05396 | 0.004575 | 4.16E-32 | NRG4 | 0.0010745 | 310.485 |
| rs12908437 | 15 | 99287375 | T | C | 0.3761 | 0.045764 | 0.0039845 | 1.56E-30 | IGF1R | 0.0009829 | 283.982 |
| rs1478604 | 15 | 39873321 | T | C | 0.7061 | -0.026249 | 0.0042092 | 4.49E-10 | THBS1 | 0.000286 | 82.568 |
| rs2472297 | 15 | 75027880 | T | C | 0.2487 | -0.027944 | 0.0049352 | 1.5E-08 | CYP1A1 | 0.0002918 | 84.254 |
| rs2929508 | 15 | 72246964 | A | T | 0.2608 | -0.028981 | 0.0049126 | 3.65E-09 | MYO9A | 0.0003238 | 93.505 |
| rs55781567 | 15 | 78857986 | C | G | 0.6548 | 0.023147 | 0.0040512 | 1.11E-08 | CHRNA5 | 0.0002422 | 69.931 |
| rs57737646 | 15 | 76299828 | T | C | 0.0252 | -0.093941 | 0.012489 | 5.4E-14 | NRG4 | 0.0004336 | 125.202 |
| rs8040109 | 15 | 73334225 | A | C | 0.7074 | 0.024914 | 0.00428 | 5.85E-09 | NEO1 | 0.000257 | 74.188 |
| rs11644696 | 16 | 81572093 | A | G | 0.4766 | 0.022064 | 0.0038899 | 1.41E-08 | CMIP | 0.0002429 | 70.123 |
| rs4788815 | 16 | 71634811 | A | T | 0.3570 | -0.026241 | 0.00403 | 7.44E-11 | TAT | 0.0003161 | 91.280 |
| rs4997081 | 16 | 20365234 | C | G | 0.1962 | -0.030192 | 0.0048328 | 4.18E-10 | UMOD | 0.0002875 | 83.014 |
| rs57652769 | 16 | 79753976 | T | C | 0.3094 | -0.036197 | 0.0042129 | 8.56E-18 | MAFTRR | 0.0005599 | 161.708 |
| rs62052820 | 16 | 69575238 | A | G | 0.2124 | 0.041401 | 0.0047484 | 2.81E-18 | MIR1538 | 0.0005735 | 165.626 |
| rs8050136 | 16 | 53816275 | A | C | 0.4029 | 0.02464 | 0.0038882 | 2.34E-10 | FTO | 0.0002921 | 84.343 |
| rs9925837 | 16 | 79927303 | A | G | 0.8445 | -0.041628 | 0.0053322 | 5.85E-15 | LINC01229 | 0.0004551 | 131.430 |
| rs2453580 | 17 | 19438321 | T | C | 0.5977 | 0.024703 | 0.0040064 | 7.01E-10 | SLC47A1 | 0.0002935 | 84.734 |
| rs3794748 | 17 | 53365172 | A | G | 0.4088 | 0.037619 | 0.0039418 | 1.38E-21 | HLF | 0.0006841 | 197.585 |
| rs9895661 | 17 | 59456589 | T | C | 0.8174 | 0.050208 | 0.0051 | 7.23E-23 | BCAS3 | 0.0007525 | 217.373 |
| rs11663816 | 18 | 57876227 | T | C | 0.7295 | -0.030377 | 0.0042881 | 1.4E-12 | MC4R | 0.0003642 | 105.157 |
| rs10405423 | 19 | 7211311 | A | C | 0.6625 | 0.03865 | 0.004143 | 1.07E-20 | INSR | 0.000668 | 192.951 |
| rs10414501 | 19 | 50259674 | C | G | 0.9570 | -0.125438 | 0.0177451 | 1.56E-12 | TSKS | 0.001295 | 374.282 |
| rs2868194 | 19 | 33350060 | T | C | 0.4084 | -0.026759 | 0.0039217 | 8.9E-12 | SLC7A9 | 0.000346 | 99.908 |
| rs35396326 | 19 | 45357003 | C | G | 0.7040 | 0.024742 | 0.0044281 | 2.31E-08 | NECTIN2 | 0.0002551 | 73.662 |
| rs4808762 | 19 | 18326222 | T | C | 0.7199 | -0.024229 | 0.0042668 | 1.36E-08 | PDE4C | 0.0002367 | 68.353 |
| rs57070985 | 19 | 4969053 | A | G | 0.6461 | 0.028568 | 0.0040629 | 2.04E-12 | KDM4B | 0.0003732 | 107.770 |
| rs62128132 | 19 | 50217955 | T | C | 0.9661 | -0.117564 | 0.0147093 | 1.32E-15 | CPT1C | 0.0009053 | 261.554 |
| rs142773928 | 20 | 43038720 | A | G | 0.1660 | 0.031688 | 0.0053763 | 3.77E-09 | HNF4A | 0.000278 | 80.275 |
| rs1800961 | 20 | 43042364 | T | C | 0.0339 | -0.075792 | 0.0118559 | 1.63E-10 | HNF4A | 0.0003763 | 108.650 |
| rs6119510 | 20 | 33287782 | T | G | 0.5959 | -0.023095 | 0.0039005 | 3.2E-09 | TP53INP2 | 0.0002569 | 74.166 |
| rs7267595 | 20 | 10643850 | A | C | 0.5097 | 0.022524 | 0.0038025 | 3.15E-09 | JAG1 | 0.0002536 | 73.211 |
| rs219781 | 21 | 37832621 | T | G | 0.2456 | -0.025151 | 0.0044479 | 1.56E-08 | CLDN14 | 0.0002344 | 67.677 |
| rs12485100 | 22 | 44325516 | T | G | 0.1726 | -0.03262 | 0.0051526 | 2.44E-10 | PNPLA3 | 0.0003039 | 87.751 |
